# Time to response for clinical and patient-reported outcomes in patients with psoriatic arthritis treated with tofacitinib, adalimumab, or placebo

**DOI:** 10.1186/s13075-022-02721-0

**Published:** 2022-02-09

**Authors:** Dafna D. Gladman, Laura C. Coates, Joseph Wu, Lara Fallon, Elizabeth D. Bacci, Joseph C. Cappelleri, Andrew G. Bushmakin, Philip S. Helliwell

**Affiliations:** 1 Department of Medicine, University of Toronto, Schroeder Arthritis Institute, Krembil Research Institute, Toronto Western Hospital, Toronto, ON Canada; 2 University of Oxford, Oxford, UK; 3 Pfizer Inc, Groton, CT USA; 4 Pfizer Inc, Kirkland, QC Canada; 5 Evidera, Seattle, WA USA; 6 Leeds Institute of Rheumatic and Musculoskeletal Medicine, University of Leeds, Leeds, UK

**Keywords:** Arthritis, Psoriatic, Anti-rheumatic agents

## Abstract

**Background:**

This study examined the time to clinically meaningful response in patients with active psoriatic arthritis treated with tofacitinib, adalimumab, or placebo switching to tofacitinib.

**Methods:**

Data were from two phase 3 studies, OPAL Broaden (12 months) and OPAL Beyond (6 months). Patients received tofacitinib 5 or 10 mg twice daily (BID), adalimumab 40 mg once every 2 weeks (OPAL Broaden only), or placebo switching to tofacitinib 5 or 10 mg BID at month 3. Baseline to initial response time was according to pre-defined clinically meaningful criteria on Health Assessment Questionnaire-Disability Index (HAQ-DI; ≥ 0.35-point improvement), Functional Assessment of Chronic Illness Therapy-Fatigue (FACIT-F; ≥ 4-point improvement), Psoriatic Arthritis Disease Activity Score (PASDAS; post-baseline score ≤ 3.2 and > 1.6-point improvement from baseline), and minimal disease activity (MDA; meeting at least 5 of 7 criteria) composite.

**Results:**

In OPAL Broaden, median time to initial HAQ-DI score response was 29, 53, and 30 days in patients treated with tofacitinib 5 mg BID, tofacitinib 10 mg BID, or adalimumab, compared with 162 and 112 days in patients treated with placebo switching to tofacitinib 5 or 10 mg BID at month 3, respectively. Across studies, median time to initial FACIT-F total score response was shorter in patients receiving tofacitinib 5 mg BID (31 days) vs other groups (84–92 days). Median time to initial response was approximately 11 (MDA)/6–9 months (PASDAS) in tofacitinib/adalimumab groups in OPAL Broaden.

**Conclusion:**

This analysis demonstrates tofacitinib’s efficacy on most patient-reported and clinical endpoints over time and shows a shorter time to initial, clinically meaningful response in patients receiving tofacitinib vs patients switching from placebo to tofacitinib.

**Trial registration:**

ClinicalTrials.gov, NCT01877668. Registered June 12, 2013. ClinicalTrials.gov, NCT01882439. Registered June 18, 2013.

## Background

Psoriatic arthritis (PsA) is a chronic systemic disease that is characterized by inflammatory arthritis, enthesitis, dactylitis, axial disease, and skin manifestations [1]. The complexity and heterogeneity of PsA often result in a diagnostic delay, which can negatively impact prognosis, in terms of peripheral joint destruction and long-term physical functioning [2, 3]. Observational data suggest a median lag time of 12 months from onset of disease and first rheumatological assessment, with some patients waiting up to 2 years for a first assessment [2]. Clinical guidelines and initiatives like the Tight Control of Psoriatic Arthritis (TICOPA) protocol prioritize the early detection and treat-to-target management of PsA disease activity, which can lead to improved outcomes for patients with respect to symptom control, health-related quality of life, and joint preservation [1, 4–8]. Achieving rapid and clinically meaningful improvement of disease severity is important for physicians and patients alike. Patients with PsA prioritize symptom alleviation (in particular, the relief of pain), reducing the impact of their disease on daily life and slowing of disease progression, all of which can be better achieved with timely diagnosis and early treatment initiation [9, 10].

Given the multifactorial nature of PsA, composite endpoints that group multiple disease domains within a single outcome measure are preferred over assessing symptom domains in isolation [11, 12]. The Psoriatic Arthritis Disease Activity Score (PASDAS) and minimal disease activity (MDA) are composite measures used to examine changes in PsA disease activity over time, and their responsivity has been demonstrated in PsA patient populations undergoing treatment with tofacitinib [11].

Tofacitinib is an oral Janus kinase inhibitor for the treatment of PsA. The efficacy and safety of oral tofacitinib 5 or 10 mg twice daily (BID) or adalimumab (40 mg administered subcutaneously once every 2 weeks [Q2W]) have been demonstrated in patients with active PsA and a previous inadequate response to conventional synthetic (cs) disease-modifying antirheumatic drugs (DMARDs) (OPAL Broaden) or tumor necrosis factor inhibitors (TNFi) (OPAL Beyond) [13, 14] and were investigated in a long-term extension study (OPAL Balance, NCT01976364) [15]. Tofacitinib was more effective than placebo over 3 months in reducing disease activity [13, 14]. Post hoc analyses of these data have shown improvements in patient-reported outcomes within 2–4 weeks of tofacitinib initiation [16, 17]. Rapid and sustained improvements in pain parameters have been observed as early as 2 weeks following initiation of tofacitinib in patients with PsA or rheumatoid arthritis [18]. However, OPAL Broaden was not designed to assess superiority or non-inferiority between tofacitinib and adalimumab; therefore, no statistical comparisons between the active treatments were made, and only numerical comparisons were provided.

The long-term success of a particular treatment for PsA may be predictable as early as 3 months following treatment initiation, emphasizing the importance of the timely assessment and adjustment of the disease management approach, if necessary, to optimize patient outcomes by reducing disease activity [19]. Moreover, the 3-month time frame is used by some regulatory systems to assess the efficacy of a drug (e.g., the National Institute for Health and Care Excellence) [20]. Clarification of the time frame over which a meaningful response to treatment can be expected, and availability of comparative data between drug classes, would be of considerable value to clinicians when considering treatment options in PsA. This post hoc analysis of data from OPAL Broaden and OPAL Beyond examined the time to a clinically meaningful response for selected patient-reported outcomes and clinical measures, in patients with active PsA treated with tofacitinib, adalimumab, or placebo switching to tofacitinib.

## Materials and methods

### Study design and patient population

Data for this post hoc time-to-response analysis were derived from two randomized, double-blind, placebo-controlled phase 3 studies in patients treated for active PsA: OPAL Broaden (*n* = 422; NCT01877668; 12 months’ duration) and OPAL Beyond (*n* = 394; NCT01882439; 6 months’ duration); both have been described previously [13, 14]. In both studies, patients were aged > 18 years (or ≥ 20 years in Taiwan) and had received a diagnosis of PsA at least 6 months previously, in line with Classification Criteria for Psoriatic Arthritis (CASPAR) [13, 14]. Depending on the study, patients had previously experienced an inadequate response to at least one prior csDMARD and were TNFi-naïve (OPAL Broaden), or had experienced an inadequate response to at least one prior TNFi (OPAL Beyond) [13, 14]. Patients were randomized to either tofacitinib 5 mg BID, tofacitinib 10 mg BID, adalimumab 40 mg Q2W via subcutaneous injection (OPAL Broaden only), or placebo switching to tofacitinib 5 or 10 mg BID at month 3. All patients received a stable dose of a single csDMARD throughout both studies. Of note, direct comparisons between adalimumab and tofacitinib were not possible, as OPAL Broaden and OPAL Beyond were not adequately powered for non-inferiority or superiority comparisons between active treatments.

### Assessments

Time (in days) from baseline to initial response was assessed based on pre-defined definitions. The Health Assessment Questionnaire-Disability Index (HAQ-DI) score response, defined as a ≥ 0.35-point improvement (decrease) from baseline (analyzed for patients with baseline HAQ-DI ≥ 0.35) [21], was measured at 0.5, 1, 2, 3, 4, and 6 months post-baseline and additionally at months 9 and 12 (OPAL Broaden only). All other outcomes were measured at months 1, 3, and 6 (both studies) and additionally at months 9 and 12 (OPAL Broaden only). The Functional Assessment of Chronic Illness Therapy-Fatigue (FACIT-F) total score response was defined as a ≥ 4-point improvement (increase) from baseline [22].

An MDA composite score response (yes/no) required a patient to meet at least 5 of the following 7 criteria: tender joint count ≤ 1, swollen joint count ≤ 1, Psoriasis Area and Severity Index (PASI) score ≤ 1 or body surface area affected ≤ 3%, Patient’s Assessment of Arthritis Pain (visual analog scale [VAS]) ≤ 15 mm, Patient’s Global Assessment of Arthritis (VAS) ≤ 20 mm, HAQ-DI score ≤ 0.5, or tender entheseal points (using the Leeds Enthesitis Index) ≤ 1 [23]. Finally, a PASDAS score response was defined as a post-baseline score of ≤ 3.2 and > 1.6-point improvement (decrease) from baseline (good clinical response), analyzed for patients with baseline PASDAS > 3.2 [24].

### Statistical analyses

Time-to-response analyses were performed separately for the OPAL Broaden (up to month 12) and OPAL Beyond (up to month 6) data sets using the Kaplan–Meier method, with patients censored at the last observed visit [25]. The median times (95% confidence interval) to initial response from baseline (in days) for the treatment groups (i.e., 50% of patients would have a response before this time, and 50% would have a response after this time) were estimated from the Kaplan–Meier analyses, when the time-to-response curves reached below the median line, otherwise median times were not estimable. The times to 25th percentile to initial response from baseline of the treatment groups were also estimated (i.e., 25% of patients would have a response before this time, and 75% would have a response after this time). Patients in the full analysis set were included and analyzed in the treatment sequences in which they were randomized.

Log-rank (Mantel–Haenszel) tests [26] were performed to compare time to initial response curves across treatment groups. Statistical significance was reported as *p* ≤ 0.05, based on a chi-square test with degrees of freedom = number of treatments – 1, without adjustment for multiple comparisons. A statistically significant result indicated that at least two treatment groups were different in their time to initial response curves. The OPAL Broaden and OPAL Beyond studies were not adequately powered for non-inferiority or superiority comparisons between active treatment groups. All analyses were conducted by Evidera (Bethesda, MD, USA).

## Results

### Patients

The time-to-response analysis was based on data from 816 patients (OPAL Broaden, *n* = 422; OPAL Beyond, *n* = 394). Baseline patient demographics and disease characteristics have been reported previously [13, 14].

### Health Assessment Questionnaire-Disability Index score response

**Fig. 1:**
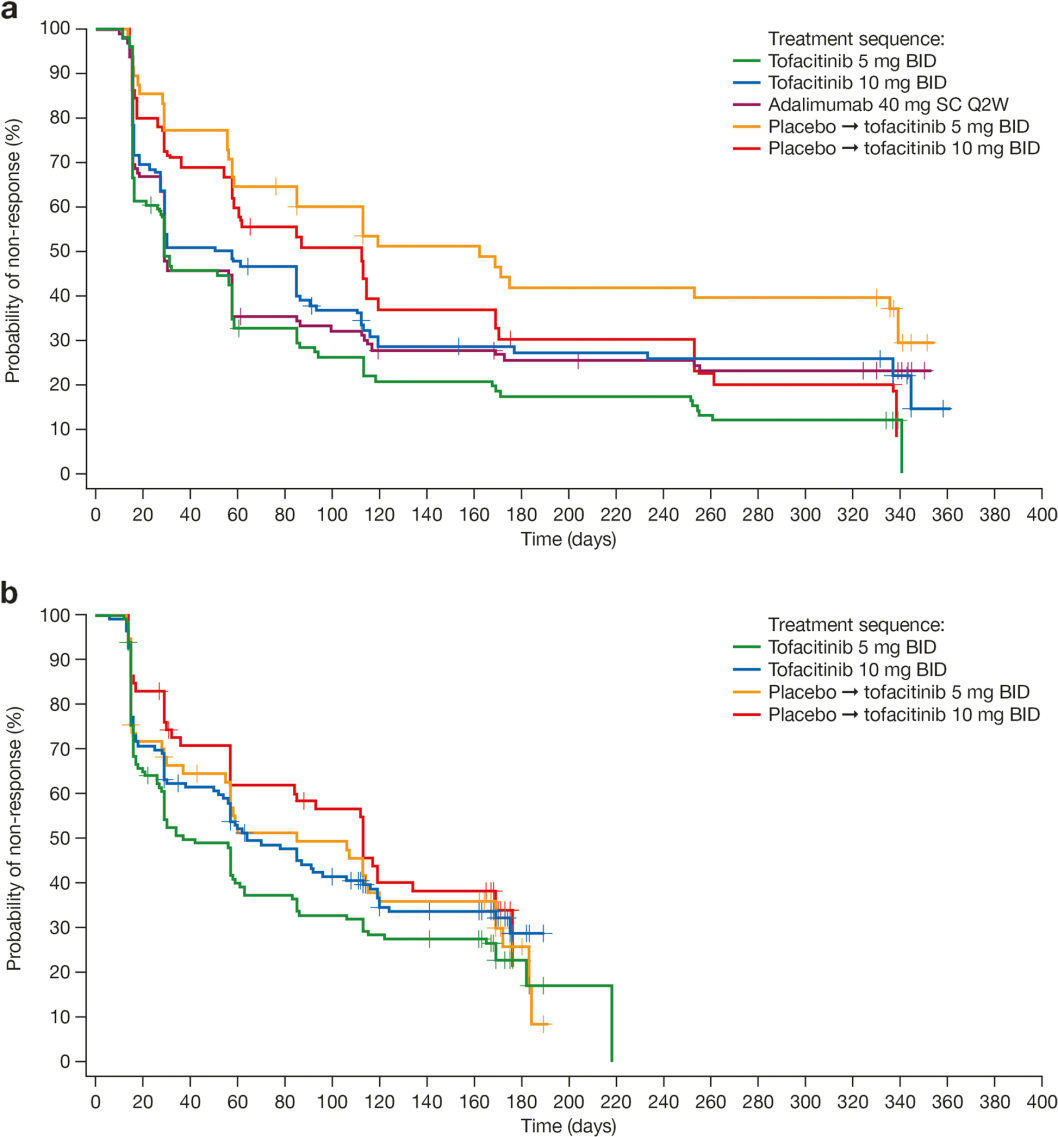
Time to initial HAQ-DI score response: **a** OPAL Broaden, **b** OPAL Beyond (FAS). Score response defined for HAQ-DI as ≥ 0.35-point improvement from baseline (analyzed for patients with baseline HAQ-DI ≥ 0.35). BID, twice daily; FAS, full analysis set; HAQ-DI, Health Assessment Questionnaire-Disability Index; Q2W, once every 2 weeks; SC, subcutaneous

In OPAL Broaden, median time to an initial HAQ-DI score response (defined as ≥ 0.35-point improvement from baseline) was shorter in patients treated with tofacitinib 5 mg BID and adalimumab (approximately 1 month [29–30 days]), and patients treated with tofacitinib 10 mg BID (53.5 days), vs patients who received placebo up to month 3 (~ 90 days) followed by tofacitinib 5 or 10 mg BID (approximately 4–5 months [162 and 112 days, respectively]) (*p* < 0.01; Table 1, Fig. 1a). In OPAL Beyond, a similar trend was observed where the median time to initial HAQ-DI score response was approximately 1 month (37 days) for tofacitinib 5 mg BID (Table 1, Fig. 1b). Across studies, the time to 25th percentile to initial HAQ-DI score response was similar between the tofacitinib 5 or 10 mg BID and adalimumab treatment groups (15–16 days) and among patients who initially received placebo for 3 months and switched to tofacitinib 10 mg BID (29–30 days) (Table 1). In patients who initially received placebo up to month 3 and switched to tofacitinib 5 mg BID, the time to 25th percentile to initial HAQ-DI score response was longer in OPAL Broaden (55 days) than in OPAL Beyond (16 days) (Table 1).

**Table 1:** Time to initial response for outcomes in OPAL Broaden and Beyond (FAS)

	OPAL Broaden				OPAL Beyond			
	Responders		Days to response		Responders		Days to response	
Response Treatment sequence	***N*** at baseline	Responders (up to month 12)	25th percentile (95% CI)	Median (95% CI)	***N*** at baseline	Responders (up to month 6)	25th percentile (95% CI)	Median (95% CI)
**HAQ-DI score response**^**a**^								
Tofacitinib 5 mg BID	96	84	15.0 (15.0–16.0)	30.0 (27.0–57.0)	116	88	16.0 (15.0–20.0)	37.0 (29.0–61.0)
Tofacitinib 10 mg BID	92	71	16.0 (15.0–27.0)	53.5 (29.0–90.0)	123	80	16.0 (15.0–29.0)	64.0 (57.0–113.0)
ADA 40 mg SC Q2W	96	73	15.0 (15.0–27.0)	29.0 (29.0–57.0)	–	–	–	–
PBO → tofacitinib 5 mg BID	48	30	55.0 (28.0–113.0)	162.0 (85.0–NE)	57	40	16.0 (15.0–57.0)	85.0 (57.0–169.0)
PBO → tofacitinib 10 mg BID	46	36	29.0 (16.0–60.0)	112.0 (57.0–169.0)	59	38	30.0 (17.0–84.0)	113.0 (84.0–169.0)
*p* value				0.0031^**^				0.1458
**FACIT-F total score response**^**b**^								
Tofacitinib 5 mg BID	107	87	29.0 (NE–NE)	31.0 (29.0–43.0)	130	98	29.0 (NE–NE)	31.0 (30.0–84.0)
Tofacitinib 10 mg BID	104	84	29.0 (29.0–30.0)	85.0 (34.0–92.0)	132	91	29.0 (NE–NE)	84.0 (32.0–91.0)
ADA 40 mg SC Q2W	106	83	29.0 (29.0–30.0)	85.0 (61.0–92.0)	–	–	–	–
PBO → tofacitinib 5 mg BID	52	42	30.5 (29.0–85.0)	86.0 (85.0–169.0)	66	49	29.0 (29.0–34.0)	85.0 (35.0–164.0)
PBO → tofacitinib 10 mg BID	53	41	29.5 (29.0–33.0)	85.0 (31.0–170.0)	65	41	29.0 (29.0–44.0)	92.0 (81.0–175.0)
*p* value				0.4136				0.1164
**MDA response**^**c**^								
Tofacitinib 5 mg BID	107	55	85.0 (85.0–253.0)	337.0 (256.0–NE)	131	46	92.0 (85.0–169.0)	NE (NE–NE)
Tofacitinib 10 mg BID	104	54	86.0 (85.0–168.0)	337.0 (171.0–NE)	132	39	96.0 (85.0–NE)	NE (NE–NE)
ADA 40 mg SC Q2W	106	53	86.0 (83.0–169.0)	339.0 (171.0–NE)	–	–	–	–
PBO → tofacitinib 5 mg BID	52	21	251.0 (169.0–339.0)	342.0 (337.0–NE)	66	17	167.0 (86.0–189.0)	189.0 (NE–NE)
PBO → tofacitinib 10 mg BID	53	25	169.0 (168.0–241.0)	338.0 (176.0–NE)	65	21	169.0 (162.0–176.0)	176.0 (174.0–NE)
*p* value				0.5962				0.6995
**PASDAS score response**^**d**^								
Tofacitinib 5 mg BID	107	67	90.0 (84.0–170.0)	253.0 (174.0–335.0)	123	49	85.0 (83.0–167.0)	NE (NE–NE)
Tofacitinib 10 mg BID	103	67	85.0 (83.0–88.0)	176.0 (166.0–258.0)	129	51	86.0 (85.0–168.0)	182.0 (169.0–NE)
ADA 40 mg SC Q2W	104	59	87.0 (85.0–169.0)	253.0 (169.0–NE)	–	–	–	–
PBO → tofacitinib 5 mg BID	52	24	170.0 (166.0–253.0)	344.0 (251.0–NE)	63	22	168.0 (85.0–173.0)	189.0 (169.0–189.0)
PBO → tofacitinib 10 mg BID	52	31	169.0 (169.0–175.0)	241.0 (170.0–323.0)	65	28	169.0 (163.0–169.0)	173.0 (169.0–190.0)
*p* value				0.2118				0.9613

Log-rank (Mantel–Haenszel) tests comparing Kaplan–Meier curves across treatments; *p* value based on chi-square test with degrees of freedom = number of treatments – 1; **p ≤ 0.01

PBO → tofacitinib 5 or 10 mg BID groups refers to blinded placebo treatment to month 3 followed by blinded switching to tofacitinib 5 or 10 mg BID

^a^HAQ-DI ≥ 0.35-point improvement from baseline (analyzed for patients with baseline HAQ-DI ≥ 0.35)

^b^FACIT-F total score ≥ 4-point improvement from baseline

^c^MDA yes/no composite response (meeting at least 5 of 7 criteria)

^d^PASDAS post-baseline score of ≤ 3.2 and > 1.6-point improvement from baseline (analyzed for patients with baseline PASDAS > 3.2)

*Abbreviations: ADA* adalimumab, *BID* twice daily, *CI* confidence interval, *FACIT-F* Functional Assessment of Chronic Illness Therapy-Fatigue, *FAS* full analysis set, *HAQ-DI* Health Assessment Questionnaire-Disability Index, *MDA* minimal disease activity, *NE* not estimable, *PASDAS* Psoriatic Arthritis Disease Activity Score, *PBO* placebo, *Q2W* once every 2 weeks, *SC* subcutaneous

### Functional Assessment of Chronic Illness Therapy-fatigue total score response

**Fig. 2:**
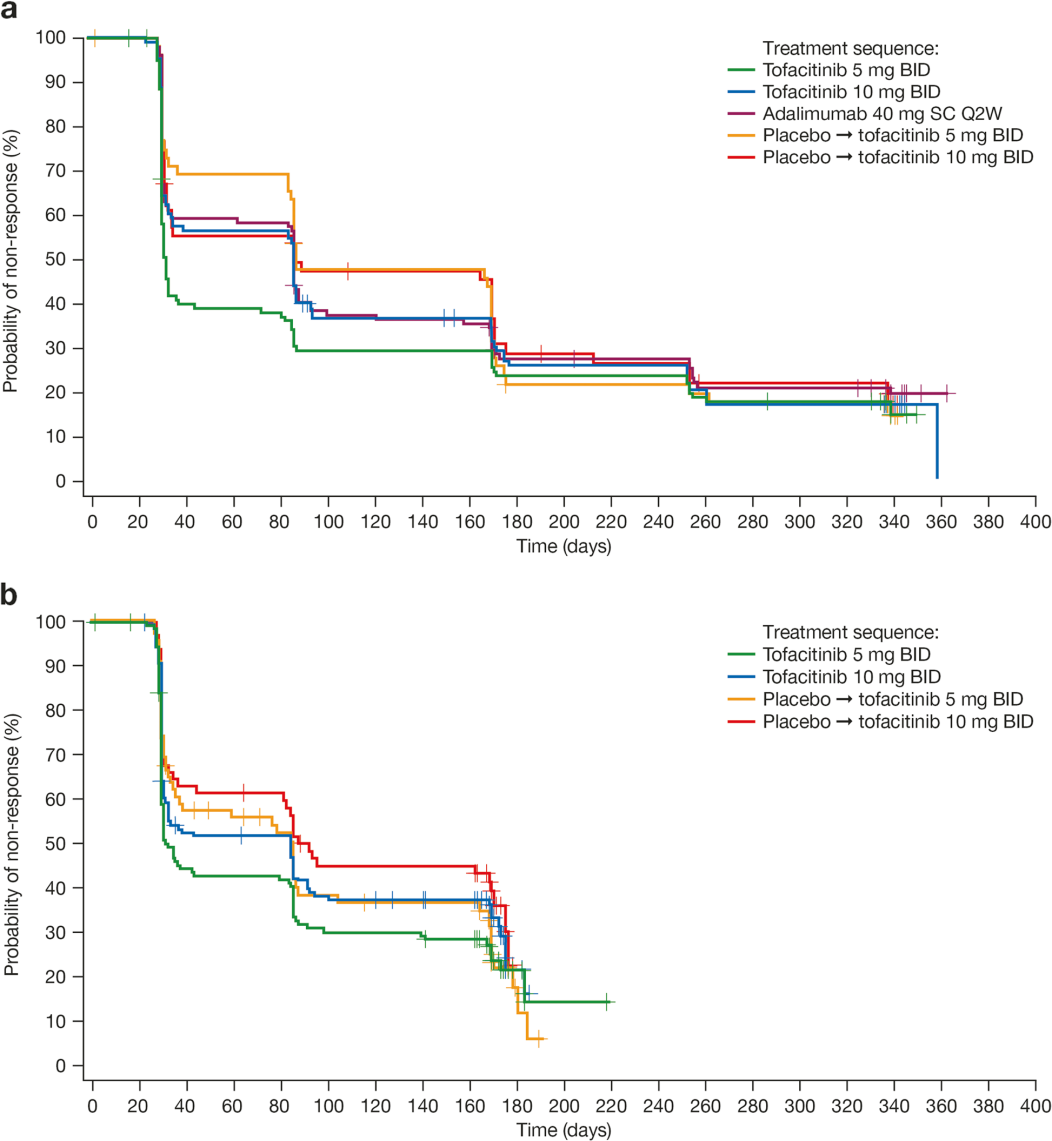
Time to initial FACIT-F total score response: **a** OPAL Broaden, **b** OPAL Beyond (FAS). Response defined for FACIT-F total score as ≥ 4-point improvement from baseline. BID, twice daily; FACIT-F, Functional Assessment of Chronic Illness Therapy-Fatigue; FAS, full analysis set; Q2W, once every 2 weeks; SC, subcutaneous

Initial FACIT-F total score responses, defined as improvement from baseline of at least 4 points, were achieved faster in patients receiving tofacitinib 5 mg BID compared with all other treatment groups in both OPAL Broaden and OPAL Beyond, although the difference between treatment groups was not significant in either study (*p* > 0.05; Table 1, Fig. 2). In both studies, the median time to initial FACIT-F total score response was approximately 1 month (31 days) in patients receiving tofacitinib 5 mg BID and approximately 3 months in all other treatment groups (84–92 days) (Table 1, Fig. 2). The time to the 25th percentile to initial FACIT-F total score response was similar for the tofacitinib 5 or 10 mg BID and adalimumab treatment groups (29 days) and among patients who initially received placebo for 3 months and switched to tofacitinib 5 or 10 mg BID (30.5 and 29.5 days, respectively) (Table 1).

### Minimal disease activity response

**Fig. 3:**
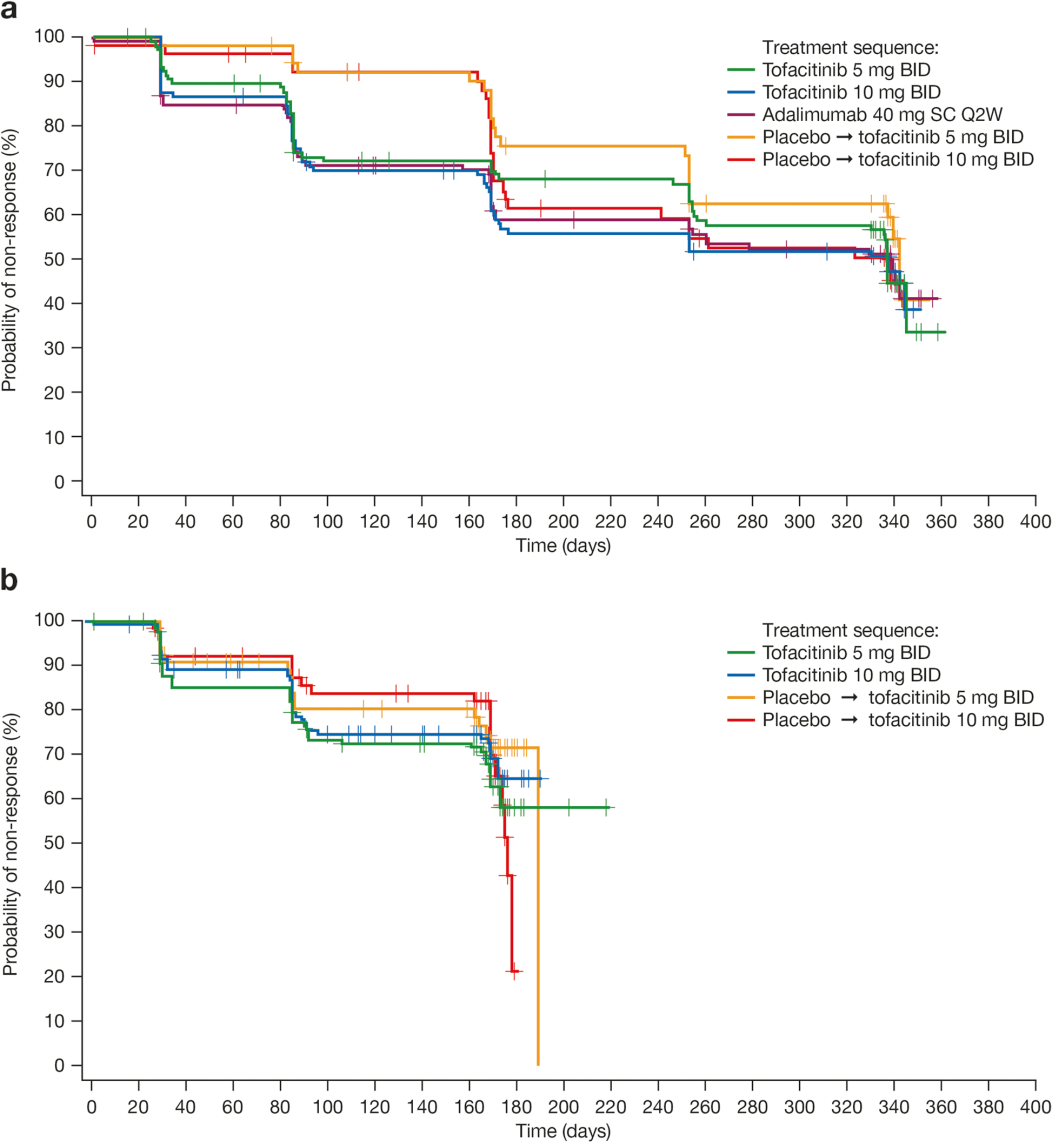
Time to initial MDA response: **a** OPAL Broaden, **b** OPAL Beyond (FAS). Response defined for MDA as meeting ≥ 5 of 7 of the following disease activity outcome criteria: tender joint count ≤ 1; swollen joint count ≤ 1; Psoriasis Area and Severity Index score ≤ 1 or body surface area ≤ 3%; Patient’s Assessment of Arthritis Pain VAS ≤ 15 mm; Patient’s Global Assessment of Arthritis VAS ≤ 20 mm; Health Assessment Questionnaire-Disability Index score ≤ 0.5; or tender entheseal points (using the Leeds Enthesitis Index) ≤ 1 [23]. BID, twice daily; FAS, full analysis set; MDA, minimal disease activity; Q2W, once every 2 weeks; SC, subcutaneous; VAS, visual analog scale

Overall, there was no statistical difference between treatment groups in time to initial MDA response in either OPAL Broaden or OPAL Beyond (*p* > 0.05; Table 1, Fig. 3). In OPAL Broaden, median times to initial MDA response in patients who initially received tofacitinib or adalimumab were approximately 11 months (337–339 days) (Table 1, Fig. 3a). Patients receiving active treatment since baseline appeared more likely to have an initial MDA response within the first 3 months, compared with patients who received placebo and switched to tofacitinib 5 or 10 mg BID at month 3 (Fig. 3a). Across studies, the time to the 25th percentile to initial MDA response was similar between the tofacitinib 5 or 10 mg BID and adalimumab treatment groups (85–96 days) and among patients who initially received placebo for 3 months and switched to tofacitinib 10 mg BID (169 days). In patients who initially received placebo and switched to tofacitinib 5 mg BID at month 3, the time to the 25th percentile to initial MDA response was longer in OPAL Broaden (251 days) than in OPAL Beyond (167 days).

### Psoriatic Arthritis Disease Activity Score response

**Fig. 4:**
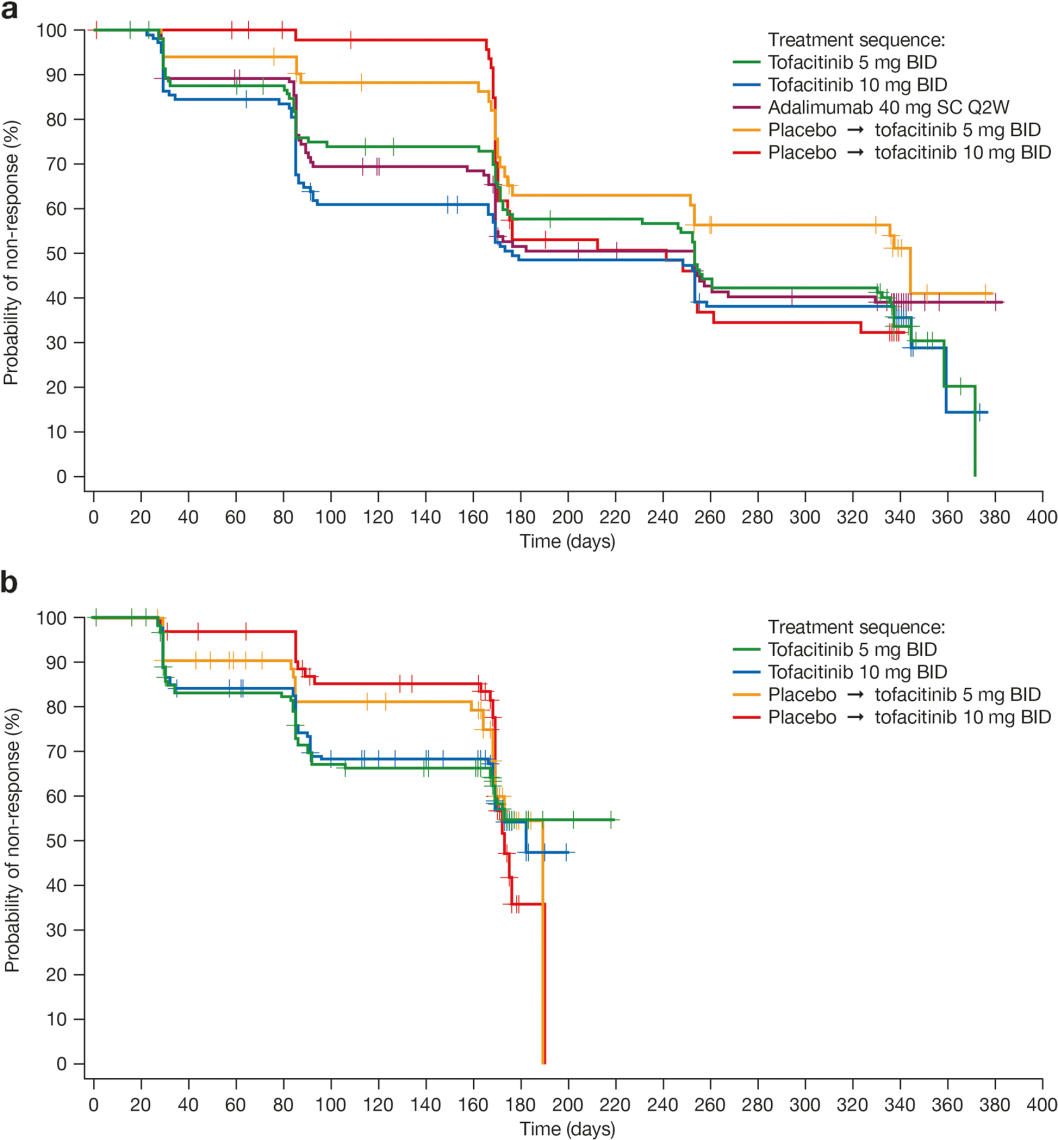
Time to initial PASDAS response: **a** OPAL Broaden, **b** OPAL Beyond (FAS). Response defined for PASDAS as post-baseline score of ≤ 3.2 and > 1.6-point improvement from baseline (analyzed for patients with baseline PASDAS > 3.2). BID, twice daily; FAS, full analysis set; PASDAS, Psoriatic Arthritis Disease Activity Score; Q2W, once every 2 weeks; SC, subcutaneous

Overall, times to initial PASDAS response (defined as a post-baseline score of ≤ 3.2 and > 1.6-point improvement from baseline) were not significantly different between treatment groups in either OPAL Broaden or OPAL Beyond (*p* > 0.05; Table 1, Fig. 4). Patients receiving tofacitinib or adalimumab (OPAL Broaden only) since baseline appeared more likely to have an initial PASDAS response within the first 3 months, compared with patients who received placebo for 3 months and switched to tofacitinib (Fig. 4). For patients receiving either tofacitinib or adalimumab in OPAL Broaden, median time to an initial PASDAS response ranged between approximately 6 and 9 months (176–253 days) (Fig. 4a). In OPAL Beyond, median times to an initial PASDAS response in patients who received tofacitinib 10 mg BID, or placebo switching to tofacitinib at month 3, were approximately 6 months (173–189 days) (Fig. 4b). Across studies, the time to the 25th percentile to initial PASDAS response after starting treatment was similar between the tofacitinib 5 or 10 mg BID and adalimumab treatment groups (85–90 days) and among patients who initially received placebo and switched to tofacitinib 5 or 10 mg BID (168–170 days) (Table 1).

## Discussion

This post hoc analysis of data from the phase 3 OPAL Broaden and OPAL Beyond studies examined the time to a clinically meaningful response for selected patient-reported outcomes (HAQ-DI and FACIT-F) and two composite measures of PsA disease activity (MDA and PASDAS), in patients with active PsA treated with tofacitinib, adalimumab, or placebo switching to tofacitinib over the course of 6 or 12 months.

Previous analyses of data from tofacitinib studies in patients with PsA have shown the emergence of responses across several clinical domains within 3 months of treatment initiation [13, 14] and statistically significant improvements in patient-reported outcomes and pain parameters within 2–4 weeks [16–18]. This is consistent with studies of other DMARDs in PsA populations (e.g., certolizumab pegol), in which disease activity and clinical response early on in the treatment process were shown to be predictive of likelihood of achieving later treatment targets [27]. Such “meaningful” responses, while not the treatment target per se, can be interpreted as an indication for the likelihood of achieving a target response further along the course of treatment [19]. Clarification of the time frame over which changes in core disease outcome variables should be judged, and then used as the basis for clinical decision-making, is therefore of practical value, particularly considering the benefits of prompt initiation and then subsequent adjustment of treatment to meet treatment goals [4, 5].

In our study, both median time and time to the 25th percentile (the periods during which 50% and 25% of patients experienced a given event, respectively) to initial response in terms of functional ability and fatigue (as determined by HAQ-DI and FACIT-F total score, respectively) and disease activity (as determined by MDA and PASDAS) were generally similar in patients with active PsA treated with either tofacitinib or adalimumab. The validity of the MDA and PASDAS composites as representative measures of patient- and physician-perceived changes in disease status and disability progression has been previously demonstrated in patients with PsA [28, 29]. For MDA, in OPAL Broaden, median time to initial response was approximately 11 months in patients who received tofacitinib or adalimumab; that is to say that 50% of patients experienced an initial MDA response during the first 11 months of treatment. For the PASDAS composite measure, median time to an initial response was faster and ranged from approximately 6–9 months for patients enrolled in OPAL Broaden. When looking at the time to the 25th percentile to initial score response, there was no such difference between the MDA and PASDAS composite measures, with patients who received tofacitinib or adalimumab taking approximately 3 months to achieve responder criteria. In general across both studies, patients treated with tofacitinib 5 mg BID achieved initial response in functional ability and fatigue more quickly than patients who received other treatments, although between-group differences were generally not significant; however, this is in contrast to the main analyses of the OPAL Broaden and OPAL Beyond data sets, in which numerical differences in the magnitude of from-baseline changes were not observed between the 5 and 10 mg BID doses of tofacitinib [13, 14]. This may have been due to differences in sample sizes or statistical methodology between analyses, or could relate to differences in tolerability between doses of tofacitinib, which further impacted sample sizes through the discontinuation and subsequent censoring of patients. Interestingly, in OPAL Broaden, patients who switched from placebo to tofacitinib 5 mg BID at month 3 took longer to respond (in terms of HAQ-DI and MDA, 25th percentile data) than patients who switched from placebo to tofacitinib 10 mg BID at month 3. This may have been a consequence of more persistent disease among these patients initially, resulting in a faster response to the higher dose of tofacitinib once patients were exposed to active treatment. Furthermore, the longer time to response of patients switching from placebo to tofacitinib, compared with those receiving tofacitinib throughout the observation period suggests that in cases of a lack of response to a csDMARD, it may be optimal to switch to an active advanced therapy without delay.

Time-to-response analyses in PsA suggest that the nature of the target domain, and the level of response expected, may influence how quickly a response can be achieved. For example, if response is characterized in terms of tender or swollen joint count, or PASI score, most patients respond to treatment with TNFi within 3 months of treatment initiation [30]. Baseline patient characteristics (in terms of higher tender joint counts and worse patient-reported outcomes) have also been shown to influence propensity to respond to treatment (in terms of the MDA composite) in PsA [31]. Therefore, time to response is expected to be impacted by the target domain, level of expected response, and patient baseline characteristics and disease activity.

This analysis was limited by several factors. Firstly, it is important to note that the time-to-response analyses were exploratory and were limited by the time frames of the original studies. OPAL Broaden and OPAL Beyond were not designed to compare time-to-response outcomes, as patients were assessed according to the protocol-determined fixed schedule of clinic visits. The limited number of visits may have meant that initial response events were missed and may have resulted in uneven Kaplan–Meier curves that were more difficult to interpret. Furthermore, placebo comparisons were only available to month 3, which is of limited value in time-to-event analyses. Direct comparisons between adalimumab and tofacitinib were not possible, as OPAL Broaden and OPAL Beyond were not adequately powered for non-inferiority or superiority comparisons between active treatments. Composite scores like MDA and PASDAS, composed of multiple domains and a variety of patient-reported outcomes and clinical outcome measures, may require observation periods in excess of 6 or 12 months (as for OPAL Beyond and OPAL Broaden, respectively) to register a clinically meaningful response. Other studies in patients with PsA receiving adalimumab or high-dose secukinumab have reported MDA response rates of between approximately 35–45% following 6–24 months of treatment [28, 32].

## Conclusion

In conclusion, this post hoc analysis of data from the OPAL Broaden and OPAL Beyond studies demonstrates the efficacy of tofacitinib 5 and 10 mg BID on various patient-reported and clinical endpoints over time and shows a shorter time to initial, clinically meaningful response in patients receiving tofacitinib vs patients switching from placebo to tofacitinib at month 3. Numerical similarity was observed between tofacitinib and adalimumab in OPAL Broaden. Our findings provide an estimate for physicians of when a clinically meaningful response can be expected with tofacitinib. Considering the limitations noted above, the results of this analysis should be considered exploratory and, therefore, future research is needed for confirmation.

## Data Availability

Upon request, and subject to review, Pfizer will provide the data that support the findings of this study. Subject to certain criteria, conditions, and exceptions, Pfizer may also provide access to the related individual de-identified participant data. See https://www.pfizer.com/science/clinical-trials/trial-data-and-results for more information.

## Abbreviations

ADA: Adalimumab
BID: Twice daily
CASPAR: Classification Criteria for Psoriatic Arthritis
CI: Confidence interval
csDMARD: Conventional synthetic disease-modifying antirheumatic drug
DMARD: Disease-modifying antirheumatic drug
FACIT-F: Functional Assessment of Chronic Illness Therapy-Fatigue
HAQ-DI: Health Assessment Questionnaire-Disability Index
MDA: Minimal disease activity
NE: Not estimable
PASDAS: Psoriatic Arthritis Disease Activity Score
PASI: Psoriasis Area and Severity Index
PBO: Placebo
PsA: Psoriatic arthritis
Q2W: Once every 2 weeks
SC: Subcutaneous
TICOPA: Tight Control of Psoriatic Arthritis
TNFi: Tumor necrosis factor inhibitor
VAS: Visual analog scale
